# Pretreatment Albumin-to-Alkaline Phosphatase Ratio Is a Prognostic Marker in Lung Cancer Patients: A Registry-Based Study of 7077 Lung Cancer Patients

**DOI:** 10.3390/cancers13236133

**Published:** 2021-12-06

**Authors:** Birgitte Sandfeld-Paulsen, Ninna Aggerholm-Pedersen, Anne Winther-Larsen

**Affiliations:** 1 Department of Clinical Biochemistry, Viborg Regional Hospital, 8800 Viborg, Denmark; 2 Department of Clinical Biochemistry, Aarhus University Hospital, 8200 Aarhus N, Denmark; birgne@rm.dk; 3 Department of Oncology, Aarhus University Hospital, 8200 Aarhus N, Denmark; ninnpede@rm.dk

**Keywords:** lung cancer, biomarker, prognostic, albumin, alkaline phosphatase, albumin-to-alkaline phosphatase ratio, survival

## Abstract

**Simple Summary:**

Since the albumin-to-alkaline phosphatase ratio (AAPR) has shown promising prognostic prediction in cancer patients, the prognostic value of the AAPR was evaluated in a large cohort of 7077 lung cancer patients. We combined patient data from the Danish Lung Cancer Registry and the clinical laboratory information system (LABKA) and showed that a low AAPR was independently associated with an inferior median overall survival in non-small cell lung cancer patients and small cell lung cancer patients. Furthermore, data indicated a level-dependent correlation between the AAPR and survival and that the AAPR added additional prognostic value to the already well-established prognostic markers in lung cancer. Therefore, if our findings are validated in the future, the AAPR should be incorporated as a factor in the general prognostication of lung cancer patients.

**Abstract:**

The albumin-to-alkaline phosphatase ratio (AAPR) is a novel promising prognostic marker in cancer patients. However, the evidence for its significance in lung cancer is scarce. Therefore, we assessed the prognostic value of the AAPR in a large cohort of lung cancer patients. Data on lung cancer patients diagnosed from January 2009 to June 2018 were extracted from the Danish Lung Cancer Registry and combined with data on the pretreatment serum AAPR level extracted from the clinical laboratory information system (LABKA). AAPR tertiles were applied as cutoffs. Cox proportional hazard models assessed the prognostic value of the AAPR. In total, 5978 non-small cell lung cancer (NSCLC) patients and 1099 small cell lung cancer (SCLC) patients were included. Decreasing AAPR level was significantly associated with declining median overall survival (OS) in NSCLC patients (medium vs. low AAPR, adjusted HR = 0.73 (95% confidence interval (CI) 0.68–0.79); high vs. low AAPR, adjusted HR = 0.68 (95% CI 0.62–0.73)) and in SCLC patients (medium vs. low AAPR, adjusted HR = 0.62 (95% CI 0.52–0.74); high vs. low, adjusted HR = 0.59 (95% CI 0.50–0.70)). In conclusion, the AAPR was an independent prognostic factor in NSCLC and SCLC patients. The correlation seems to be level dependent, with reducing survival found to be associated with decreasing AAPR level.

## 1. Introduction

Lung cancer is the most commonly diagnosed cancer type and the leading cause of malignancy-related death worldwide [1]. Despite several advances in treatment modalities during recent years, the prognosis of lung cancer patients remains poor. To prolong the survival, an improved stratification of lung cancer patients based on their prognosis is needed to secure a more optimal treatment strategy for each patient. The tumor node metastasis (TNM) staging system [2] and the performance stage (PS) assessment [3] are well-established and valuable prognostic markers in lung cancer. However, they fail to accurately predict the prognosis of lung cancer patients; therefore, it is of utmost importance to identify additional prognostic markers.

Albumin is the most abundant protein in the blood and its concentration in blood reflects protein status and the function of internal organs [4]. The synthesis of albumin is suppressed by inflammation and malnutrition [5], and therefore serum albumin concentration can reflect both the systemic inflammatory response in a patient [5,6] and can be used as a simple indicator of a patient’s nutritional condition [5]. In cancer patients, serum albumin level is often normal or only slightly decreased in the early stages of cancer but drops as the disease progresses, and low serum albumin levels have been found to provide negative prognostic significance in cancer patients [7]. Serum alkaline phosphatase is another marker that has been associated with prognostic impact on a cancer patient’s survival [8,9]. Alkaline phosphatase is a hydrolase enzyme concentrated in the liver, bile duct, and kidneys [10] and, in cancer patients, elevated serum levels are primarily observed when cancer involves the bone or the liver.

A new prognostic index has been introduced that combines serum albumin and serum alkaline phosphatase, i.e., the albumin-to-alkaline phosphatase ratio (AAPR), and has been shown to have significant prognostic value in several types of cancer, especially in hepatocellular carcinoma [11,12]. Interestingly, the data indicate that combining these two prognostic markers can offer additional prognostic value as compared with the single markers [13,14]. Thus, the AAPR could be a valuable marker for identifying cancer patients with inferior prognoses requiring more intensive treatment.

However, the AAPR has been evaluated only in lung cancer patients in a limited number of studies [13,15,16,17,18,19,20,21], and therefore the evidence in lung cancer is scarce. In addition, the AAPR has solely been examined in lung cancer patients of Chinese origin and in studies including restricted sample sizes. Therefore, this study aimed to evaluate the prognostic value of the pretreatment AAPR in a large cohort of Danish lung cancer patients.

## 2. Results

### 2.1. Patients

**Figure 1:**
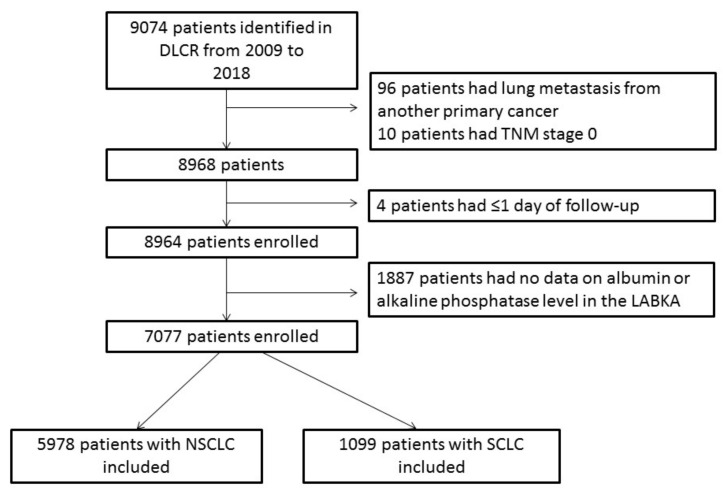
Flow chart of inclusion and exclusion of patients. Abbreviations: DLCR, Danish Lung Cancer Group; TNM, tumor node metastasis; LABKA, clinical laboratory information system; NSCLC, non-small cell lung cancer; SCLC, small cell lung cancer.

In total, 9074 patients were identified in the DLCR (Figure 1), although 110 patients were excluded as they did not have primary lung cancer (*N* = 96), had stage 0 disease (*N* = 10), or had only one day of follow-up (*N* = 4). Among the remaining 8964 lung cancer patients, serum albumin and/or serum alkaline phosphatase measurements were not available in 1887 patients, and therefore these patients were excluded. Finally, 7077 lung cancer patients were included in this study, of whom 5978 patients had non-small cell lung cancer (NSCLC) and 1099 patients had small cell lung cancer (SCLC).

Patient characteristics for included and excluded patients are shown in Table S1. The 1887 lung cancer patients excluded due to missing serum albumin and/or alkaline phosphatase measurements had a significantly higher median age, lower TNM stage, and higher PS than the 7077 included patients.

### 2.2. Patient Characteristics and AAPR Level in Patients with NSCLC

The median age of all 5978 patients with NSCLC was 70 years (5–95% percentiles, 52–84 years) (Table 1). The majority of patients were males (52%), had adenocarcinoma (53%), an advanced stage of disease (III + IV, 65%), a good PS (PS 0–1, 66%), and were current or former smokers (74%).

Patients were divided into tertiles according to their AAPR level as follows: low (AAPR < 0.3485), medium (AAPR = 0.3485–0.5067), and high (AAPR > 0.5067). In the high AAPR group, a significantly higher number of women was found than in the low and medium AAPR groups (52% vs. 46% and 46%, *p* < 0.001). Furthermore, a higher frequency of patients with a high stage of disease was found in the low group as compared with the medium and high groups, respectively (III + IV, 73% vs. 67% and 58%, *p* < 0.001) and similarly a higher frequency of patients with a poor PS was found in the low group as compared with the medium and high groups (2–4, 32% vs. 21% and 19%, *p* < 0.001).

### 2.3. Survival According to AAPR Level in Patients with NSCLC

The median overall survival (OS) of all NSCLC patients was 0.89 years (95% confidence interval (CI), 0.85–0.93 years). On the last follow-up date, 4924 patients (82%) were dead. For the remaining 1054 patients still alive, the median follow-up time was 4.24 years (5–95% percentiles, 2.11–9.97 years)

**Figure 2:**
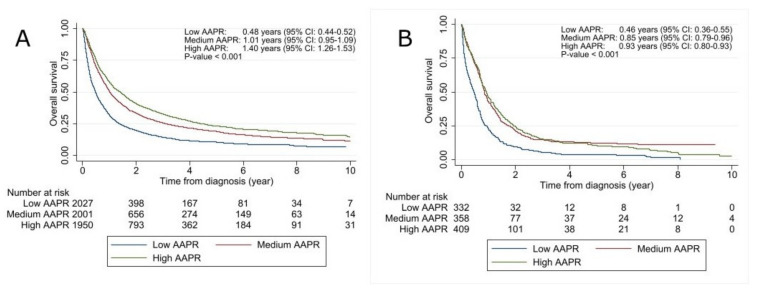
Kaplan-Meier curves of overall survival (OS) according to pretreatment AAPR level for patients with non-small cell lung cancer and small cell lung cancer: (**A**) OS according to AAPR level in non-small cell lung cancer. Patients were divided by applying tertiles (low AAPR < 0.3485, medium AAPR 0.3485–0.5067, and high AAPR > 0.5067); (**B**) OS according to AAPR level in small cell lung cancer. Patients were divided into tertiles (low AAPR < 0.3483, medium AAPR 0.3483–0.5067, and high AAPR > 0.5067). The log-rank test estimated differences between groups. AAPR, albumin-to-alkaline phosphatase ratio; CI, confidence interval.

As seen in Figure 2A, the AAPR was significantly correlated to survival with a decreasing median OS found to be associated with decreasing AAPR level: low AAPR, median OS 0.48 years (95% CI, 0.44–0.52 years); medium AAPR, 1.01 years (95% CI, 0.95–1.09 years); high AAPR, 1.40 years (95% CI, 1.26–1.53 years), *p* < 0.001. In the univariate analyses, AAPR, sex, histology, stage, age, smoking, and PS were all significantly correlated to OS (Table 2). An adjustment for potential confounders was performed to examine the independent prognostic value of the AAPR. After adjusting for sex, histology, stage, age, smoking, and PS, the AAPR remained an independent predictor of OS, and a clear level-dependent correlation was found with both the medium AAPR group (adjusted HR = 0.73 (95% CI, 0.68–0.79)) and the high AAPR group (adjusted HR = 0.68 (95% CI, 0.62–0.73)) showing increased median OS as compared with the low AAPR group (Table 2).

C-statistics were conducted to investigate if the addition of the AAPR to the existing well-established prognostic markers, stage, histology, age, sex, PS, and smoking, actually improved the prognostic value of NSCLC patients. As seen in Table 3, the prognostic model improved by the addition of the AAPR, and this improvement was statistically significant (*p* < 0.0001).

### 2.4. Patient Characteristics and AAPR Level in Patients with SCLC

For patients with SCLC, the median age was 69 years (5–95% percentiles, 52–82 years), and equal distribution of males and females was seen (Table 4). Most of the patients had an advanced stage of disease (III + IV, 85%), a good PS (0–1, 62%), and were current or former smokers (79%).

The cutoffs for the AAPR groups in SCLC patients were the following: low (AAPR < 0.3483), medium (AAPR = 0.3483–0.5067), and high (AAPR > 0.5067) groups. As compared with the medium and the high groups, the low group had a significantly higher frequency of males (59% vs. 47% and 45%, *p* < 0.001), patients with stage IV disease (70% vs. 53% and 54%, *p* < 0.001), and a poor PS (2–4, 38% vs. 26% and 26%, *p* < 0.001).

### 2.5. Survival According to AAPR in Patients with SCLC

The median OS of all SCLC patients was 0.74 years (95% CI, 0.70–0.79 years). In total, 1016 of the 1099 patients (92%) were dead at the last follow-up date. The median follow-up time of the 83 patients still alive at the last follow-up date was 5.57 years (5–95% percentiles, 2.41–9.79 years).

Similarly, for NSCLC patients, the AAPR was significantly correlated to the median OS in SCLC patients with decreasing survival found to be associated with decreasing AAPR level: low AAPR, median OS 0.46 years (95% CI, 0.36–0.55 years); medium AAPR, median OS 0.85 years (95% CI, 0.79–0.96 years); and high AAPR, median OS 0.93 years (95% CI, 0.80–1.03 years), *p* < 0.001 (Figure 2B). The univariate analyses showed that AAPR, sex, stage, age, and PS correlated with OS (Table 5). Again, the independent value of AAPR was evaluated by adjusting for the potential confounders sex, stage, age, smoking, and PS, and the AAPR remained an independent predictor of OS with an increased survival seen in the medium (adjusted HR = 0.62 (95% CI, 0.52–0.74)) and the high group (adjusted HR = 0.59 (95% CI, 0.50–0.70)) as compared with the low group (Table 5).

Once more, the C-statistics showed a significantly improved prognostic model with the inclusion of AAPR (*p* < 0.0001) (Table 3).

## 3. Discussion

This registry-based study combined data from the Danish Lung Cancer Registry and the clinical laboratory system LABKA and evaluated the new prognostic index of AAPR in a large cohort of lung cancer patients. In 5978 NSCLC patients and 1099 SCLC patients, we found that a low AAPR was independently associated with decreased survival in NSCLC as well as in SCLC patients and that there seemed to be a level-dependent correlation between AAPR level and survival, especially in NSCLC patients. In addition, we showed that the AAPR added additional prognostic value to the already well-established prognostic markers. Hence, the AAPR could improve the stratification of lung cancer patients and refine the identification of patients with poor prognoses demanding a more aggressive treatment. As far as we know, this is the most extensive study to evaluate the AAPR in lung cancer patients and the first of its kind in lung cancer patients of Caucasian ethnicity.

The AAPR was derived in 2015 by Chan et al., who investigated its prognostic value in hepatocellular cancer patients [14]. They found the ratio to hold independent prognostic value for survival and that the AAPR had improved prognostic value as compared with albumin and alkaline phosphatase alone. Studies have followed evaluating the AAPR in several other cancer types [22,23,24]. In 2019, Li et al. [16] were the first to report an association between pretreatment levels of the AAPR and survival in lung cancer patients, as they investigated 290 NSCLC patients with stage IV disease and showed that patients with an AAPR > 0.36 had independently prolonged survival with an estimate comparable with ours (adjusted HR = 0.657 (95% CI, 0.504–0.856)). Later, further studies in NSCLC patients have followed patients with advanced [13,20] and localized stages of the disease [17,19], and all studies have confirmed a significant correlation between high pretreatment AAPR and prolonged survival. However, the AAPR cutoff for defining high and low AAPR has varied significantly in these studies as the optimal cutoff is yet to be defined. Studies have used either a receiver operating characteristic curve [15,18,19] or the “Cut off Finder Software” [16,17] for identifying the optimal cutoff based on the outcome in their patient cohort, resulting in very different cutoffs varying from 0.36 to 0.64. Since applying outcome-associated cutoffs can lead to a tremendous risk of overestimating a potential association, instead, we divided patients by applying tertiles to evaluate if a level-dependent correlation between pretreatment levels of the AAPR level and OS existed. This was in line with Zhou et al. [20], who also applied tertiles for division of AAPR in 808 advanced NSCLC patients, which resulted in cutoffs very similar to ours (low < 0.34, medium 0.34–0.47, and high > 0.47). Furthermore, they found estimates very comparable with us (medium vs. low, HR 0.77 (95% CI 0.58–1.03) and high vs. low, HR 0.59 (95% CI 0.45–0.78)) supporting our finding of a level-dependent correlation.

In SCLC, previous studies have shown more divergent results [15,18,21]. In line with us, Zhou et al. [21] found a decreased OS in patients with a pretreatment AAPR < 0.35 as compared with patients with a pretreatment AAPR > 0.35 (adjusted HR = 1.65 (95% CI 1.11–2.46)) in 224 SCLC patients with extended disease. However, a correlation between pretreatment levels of the AAPR and OS could not be confirmed in a study evaluating 300 SCLC patients with extended disease [15] or in a study of 122 SCLC patients with limited disease [18]. In contrast, these two studies both found that post-treatment AAPR was prognostic for survival.

Altogether, our results confirm the previous finding of an independent prognostic value of pretreatment AAPR in NSCLC as well as SCLC patients. In addition, as all previous studies have been conducted in patients of Chinese origin, our study is the first to demonstrate that this correlation also exists in patients of Caucasian origin.

In patients with low AAPR, a higher frequency of patients was males with a poorer PS and a high stage of disease than patients with a medium or high AAPR. This indicates that patients with low AAPR generally have more advanced disease and that the AAPR could indicate the general disease severity in patients. However, when we adjusted for potential confounders, we observed that the association between low AAPR and inferior survival was independent of other known prognostic factors, demonstrating that low AAPR by itself is an adverse prognostic factor. Moreover, an improved model was reached by adding the AAPR to a prognostic model for survival in NSCLC and SCLC patients. Nonetheless, it is, of course, difficult to define if a low AAPR contributes directly to the inferior survival or if it instead is an indicator of underlying comorbidity.

AAPR is derived from the serum levels of albumin and alkaline phosphatase. In contrast to genetic and immunohistochemically biomarkers, the AAPR is a very simple and inexpensive marker obtained from peripheral blood samples which can easily be continuously monitored. Albumin is a protein synthesized in the liver and secreted into the blood, where it is the most abundant protein [4]. Its serum level has primarily been used as a simple way to assess a patient’s nutritional status and liver synthesis ability. Nevertheless, albumin is also known to be a negative acute-phase protein, as its synthesis is suppressed by systemic inflammation [25]. Accordingly, the serum albumin level can reflect several underlying conditions. Alkaline phosphatase is a hydrolase enzyme expressed in tissues throughout the body but with predominantly high levels in the liver, bile duct, and kidneys [10]. Hence, serum alkaline phosphatase is primarily affected by conditions related to the liver, bone, and kidneys, and in cancer patients, serum alkaline phosphatase is often used to screen for bone and liver metastasis. Decreased serum albumin levels and elevated alkaline phosphatase levels alone have previously been found to comprise negative prognostic value in lung cancer [26,27,28]. However, the combination of the two markers in the AAPR seems to hold improved prognostic value as compared with the markers alone [13,14], which may be explained by APPR’s reflection of the unfavorable effects of both markers, and thereby the identification of additional patients with poor prognosis.

Our study has several strengths. To the best of our knowledge, we have, by far, included the largest sample size in lung cancer. The enormous size of our cohort as compared with the previous studies, which included a maximum of 808 NSCLC patients and 300 SCLC patients, highlights the strength of our data as compared with former studies. The high completeness of the Danish registries and the possibility to link data from different registries at an individual level allows the inclusion of all patients diagnosed in a well-defined geographical area. This eliminates potential selection bias and increases the applicability of our data. Yet, despite the strengths of our study, there are some limitations to consider. Firstly, we could not exclude patients with known liver, kidney, or bone disease as these data were unavailable, and we were unable to determine if comorbidities affecting these organs could influence our data. Moreover, we did not have data on patients’ nutritional statuses and could not adjust for this in our analyses. Furthermore, no data on genetic driver mutations such as the epidermal growth factor receptor (EGFR) and the echinoderm microtubule-associated protein-like-anaplastic lymphoma kinase (EMLA-ALK) were available in NSCLC patients, and therefore we were unable to evaluate if the mutation-positive patients were evenly distributed between the three AAPR groups. Otherwise, this could have influenced the survival of the groups. Additionally, we did not have a validation cohort available to confirm our findings, and our data must be validated in an independent cohort in a future study. Lastly, we observed that the excluded patients had higher median age, lower TNM stage, and higher PS than the included patients. Hence, we cannot exclude a potential selection bias due to this difference.

## 4. Materials and Methods

### 4.1. Patients

The cohort has previously been described [29]. In brief, lung cancer patients were identified in the Danish Lung Cancer Registry (DLCR), covering more than 90% of all lung cancer patients diagnosed in Denmark [30]. Patients were included if they were registered in DLCR between 1 January 2009 and 26 June 2018 and, simultaneously, were registered in The Central Denmark Region (region covering 1,327,410 inhabitants equivalent to 23% of the Danish population [31]). The following data were retrieved from the DLCR on each patient at the time of diagnosis: sex, age, Eastern Cooperative Oncology Group (ECOG) PS, TNM stage, and smoking status. Information on tumor characteristics was provided from The national Danish Pathology Data Bank [32]. Serum albumin and serum alkaline phosphatase levels were retrieved from the clinical laboratory information system [33], which contains blood samples from hospitalized patients and patients from primary care submitted for analyses in The Central Denmark Region. Mortality data were retrieved from the Danish Civil Registration System [34]. Information on each patient was combined using the CPR number, a unique ten-digit number given to all inhabitants in Denmark at birth and residents upon immigration.

The diagnostic work-up and staging of lung cancer patients were performed according to the international guideline at the current time.

### 4.2. The Albumin-to-Alkaline Phosphatase Ratio

Pretreatment serum albumin and serum alkaline phosphatase levels were extracted on each patient from the clinical laboratory information system (LABKA). Only measurements performed up to 90 days before the lung cancer diagnosis were retrieved. If more than one measurement were available in the 90 days, the closest measurement to the diagnosis was extracted. Patients with missing data on any of the two measurements were excluded. The AAPR was calculated by dividing the serum albumin level by the serum alkaline phosphatase level. As the optimal cutoff point for AAPR is undetermined, the AAPR was evaluated by dividing into tertiles.

### 4.3. Ethics

The study was approved by the Danish Patient Safety Authority (no. 31-1521-400) and the Danish Data Protection Agency (no. 1-16-02-909-17). According to Danish legislation, register-based studies do not need approval by the regional committee on health-research ethics.

### 4.4. Statistical Analyses

Patient characteristics are given as either numbers and percentages or as median values with 5% and 95% percentiles. The Chi-square test or the rank-sum test were used to evaluate differences in patient characteristics.

Follow-up time and OS were defined as the time from diagnosis until the death due to any cause or the last follow-up date (1 July 2020). Patients still alive on the last day of follow-up were censored. If patients had an OS of merely one day, they were excluded. Since the last patients were included in the study in June 2018, all patients were followed for a minimum of two years. OS was the primary endpoint. Median OS was estimated by the Kaplan-Meier method and compared by the log-rank test. Crude and adjusted hazard ratios (HR) were calculated by the Cox proportional hazards model. Confounders were analyzed as categorical variables, except for age which was analyzed as a continuous variable.

To calculate whether the inclusion of the AAPR to the well-established prognostic markers (TNM stage, histology, age, sex, PS, and smoking) added additional prognostic value, C-statistics, in terms of Akaike’s information criteria (AIC) and Harrell’s concordance index (C-index), were calculated on models including and excluding the AAPR. For AIC, the model with the minimum AIC had the most precise prediction of OS, and only a difference of 2 or more (arbitrary values) was considered an actual difference. For the C-index, values ranged between 0.5 and 1.0, and a value of 1.0 was defined as the perfect fit. Likelihood-ratio tests were used to evaluate whether the added value was significant. All *p*-values were two-sided. All statistical analyses were performed with the Stata software version 15.1 (Stata Corporation, College Station, TX, USA).

## 5. Conclusions

In conclusion, we showed that the AAPR was an independent prognostic factor in NSCLC and SCLC patients. The correlation seems to be level dependent, as decreasing survival was associated with decreasing AAPR level, especially in NSCLC patients. Furthermore, the AAPR seems to add extra prognostic value to the already well-established prognostic factors and could improve the stratification of lung cancer patients leading to a more differentiated treatment strategy. Therefore, if our findings are validated in the future, the AAPR should be incorporated as a factor in the general prognostication of lung cancer patients.

## Figures and Tables

**Table 1:** Patient characteristics in non-small cell lung cancer patients stratified by albumin-to-alkaline phosphatase ratio at time of diagnosis (*N* = 5978).

Characteristics	All Patients	Low AAPR ^a^	Medium AAPR ^a^	High AAPR ^a^	*p*-Value ^b^
	*N* (%)	*N* (%)	*N* (%)	*N* (%)	
Total number of patients	5978	2027	2001	1950	
Age, years					0.263
Median age (5–95% percentiles)	70 (52–84)	70 (52–84)	70 (52–84)	70 (52–84)	
Sex					<0.001
Female	2874 (48)	931 (46)	924 (46)	1019 (52)	
Male	3104 (52)	1096 (54)	1077 (54)	931 (48)	
Histology					0.005
Adenocarcinoma	3197 (53)	1064 (52)	1055 (53)	1078 (55)	
Squamous cell	1424 (24)	472 (23)	528 (26)	424 (22)	
Other	1067 (18)	395 (19)	318 (16)	354 (18)	
Unknown	290 (5)	96 (5)	100 (5)	94 (5)	
Stage					<0.001
I	1067 (18)	226 (11)	351 (18)	490 (25)	
II	512 (9)	160 (8)	171 (9)	181 (9)	
III	1165 (19)	341 (17)	443 (22)	381 (20)	
IV	2775 (46)	1136 (56)	901 (45)	738 (38)	
Unknown	459 (8)	164 (8)	135 (7)	160 (8)	
Performance status, ECOG					<0.001
0	2013 (34)	522 (26)	755 (38)	736 (38)	
1	1906 (32)	632 (31)	639 (32)	635 (33)	
2	732 (12)	297 (15)	232 (12)	203 (10)	
3 + 4	704 (12)	351 (17)	182 (9)	171 (9)	
Unknown	623 (10)	225 (11)	193 (9)	205 (11)	
Smoking status					0.775
Never	281 (5)	91 (4)	92 (5)	98 (5)	
Current or former	4437 (74)	1456 (72)	1521 (76)	1460 (75)	
Unknown	1260 (21)	480 (24)	388 (19)	392 (20)	

AAPR, albumin-to-alkaline phosphatase ratio; ECOG, Eastern Cooperative Oncology Group; N, number; ^a^ AAPR divided by tertiles into low (AAPR < 0.3485), medium (AAPR 0.3485–0.5067), and high (AAPR > 0.5067) groups; ^b^ *p*-value calculated by the Chi-square test or the rank-sum test.

**Table 2:** Univariate and multivariate analyses of overall survival in non-small cell lung cancer patients (*N* = 5979).

Characteristics	HR	Adjusted HR
	(95% CI)	(95% CI)
AAPR		
Low	1	1
Medium	0.63 (0.59–0.68)	0.73 (0.68–0.79)
High	0.53 (0.50–0.57)	0.68 (0.62–0.73)
Sex		
Female	1	1
Male	1.18 (1.13–1.26)	1.20 (1.12–1.28)
Histology		
Adenocarcinoma	1	1
Squamous cell	1.49 (1.37–1.63)	1.20 (1.09–1.34)
Other	1.04 (0.97–1.11)	0.93 (0.86–1.01)
Stage		
I	1	1
II	1.39 (1.21–1.659)	1.31 (1.13–1.53)
III	2.86 (2.57–3.18)	2.96 (2.63–3.32)
IV	5.86 (5.32–6.44)	5.95 (5.34–6.62)
Age	1.02 (1.02–1.02)	1.02 (1.01–1.02)
Smoking		
Never	1	1
Current or former	1.31 (1.14–1.51)	1.52 (1.32–1.75)
Performance status, ECOG		
0	1	1
1	1.71 (1.60–1.84)	1.42 (1.32–1.53)
2	2.67 (2.44–2.93)	2.14 (1.94–2.36)
3	4.44 (4.04–4.87)	3.79 (3.39–4.23)

AAPR, albumin-to-alkaline phosphatase ratio; CI, confidence interval; ECOG, Eastern Cooperative Oncology Group; HR, hazard ratio.

**Table 3:** Predictive accuracies of the prognostic models.

Model NSCLC	AIC ^a^	C-Index ^b^
AAPR + stage + histology + age + sex + PS + smoking	57746	0.7545
Stage + histology +age + sex + PS + smoking	57877	0.7480
**Model SCLC**		
AAPR + stage + age + sex + PS + smoking	9063	0.7301
Stage + age + sex + PS + smoking	9097	0.7227

AAPR, albumin-to-alkaline phosphatase ratio; AIC, Akaike’s information criterion; NSCLC, non-small cell lung cancer; PS, performance status; SCLC, small cell lung cancer; ^a^, AIC assesses each model’s quality relative to the other model, and the model showing the minimum AIC is the model with the most optimal fit of data. The values obtained are arbitrary; ^b^, Harrell’s concordance index, C-index provides an estimate of goodness of fit for the model, and 1.0 is the perfect fit. Values vary between 0.5–1.0.

**Table 4:** Patient characteristics in small cell lung cancer patients stratified by albumin-to-alkaline phosphatase ratio at time of diagnosis (*N* = 1099).

Characteristics	All Patients	Low AAPR ^a^	Medium AAPR ^a^	High AAPR ^a^	*p*-Value ^b^
	*N* (%)	*N* (%)	*N* (%)	*N* (%)	
Total number of patients	1099	332	358	409	
Age, years					0.568
Median age (5–95% percentiles)	69 (52–82)	69 (51–83)	69 (52–82)	70 (53–83)	
Sex					<0.001
Female	551 (50)	137 (41)	189(53)	225 (55)	
Male	548 (50)	195 (59)	169 (47)	184 (45)	
Stage					<0.001
I	51 (5)	10 (3)	22 (6)	19 (5)	
II	28 (2)	2 (1)	10 (3)	16 (4)	
III	294 (27)	53 (16)	113 (31)	128 (31)	
IV	644 (58)	231 (70)	190 (53)	223 (54)	
Unknown	82 (8)	36 (11)	23 (6)	23 (6)	
Performance status, ECOG					<0.001
0	296 (27)	67 (20)	108 (30)	121 (29)	
1	381 (35)	96 (29)	134 (38)	151 (37)	
2	176 (16)	64 (19)	52 (15)	60 (15)	
3 + 4	148 (13)	63 (19)	39 (11)	46 (11)	
Unknown	98 (9)	42 (13)	25 (6)	31 (8)	
Smoking status					0.106
Never	9 (1)	0 (0)	3 (1)	6 (1)	
Current or former	861 (79)	247 (74)	287 (81)	327 (80)	
Unknown	229 (20)	85 (26)	66 (18)	78 (19)	

AAPR, albumin-to-alkaline phosphatase ratio; ECOG, Eastern Cooperative Oncology Group; N, number; ^a^, AAPR was divided by tertiles into low (AAPR < 0.3483), medium (AAPR 0.3483–0.5067), and high (AAPR > 0.5067) groups; ^b^, *p*-value calculated by the Chi-square test or the rank-sum test.

**Table 5:** Univariate and multivariate analyses of overall survival in SCLC patients (*N* = 1099).

Characteristics	HR	Adjusted HR
	(95% CI)	(95% CI)
AAPR		
Low	1	1
Medium	0.54 (0.46–0.63)	0.62 (0.52–0.74)
High	0.54 (0.47–0.63)	0.59 (0.50–0.70)
Sex		
Female	1	1
Male	1.12 (1.00–1.27)	1.13 (0.98–1.30)
Stage		
I	1	1
II	1.05 (0.55–2.00)	1.11 (0.55–2.24)
III	3.09 (2.07–4.60)	3.33 (2.15–5.16)
IV	6.47 (4.37–9.59)	6.65 (4.31–10.27)
Age	1.04 (1.03–1.04)	1.02 (1.01–1.03)
Smoking		
Never	1	1
Current or former	0.90 (0.47–1.74)	0.68 (0.35–1.32)
Performance status, ECOG		
0	1	1
1	1.51 (1.29–1.78)	1.28 (1.07–1.53)
2	2.88 (2.37–3.51)	2.29 (1.83–2.85)
3	4.15 (3.37–5.11)	4.33 (3.39–5.54)

AAPR, albumin-to-alkaline phosphatase ratio; CI, confidence interval; ECOG, Eastern Cooperative Oncology Group; HR, hazard ratio; SCLC, small cell lung cancer.

## Data Availability

In this study, publicly archived datasets were achieved from the Danish Lung Cancer Registry (DLCR) (www.lungecancer.dk) (accessed on 6 September2018), The Pathology Data Bank (www.pato-bank.dk) (accessed on 29 May 2018), the clinical laboratory information system (LABKA) (www.auh.dk/om-auh/afdelinger/blodprover-og-biokemi) (accessed on 24 February 2020), and the Danish Civil Registration System (www.sundhedsdatastyrelsen.dk) (accessed on 24 February 2020).

## Supplementary Materials

The following are available online at https://www.mdpi.com/article/10.3390/cancers13236133/s1, Table S1: Patient characteristics of included and excluded patients.
