# Supplementary material for: Pretreatment Albumin-to-Alkaline Phosphatase Ratio Is a Prognostic Marker in Lung Cancer Patients: A Registry-Based Study of 7077 Lung Cancer Patients
Source: Cancers (Basel). 2021 Dec 6;13(23):6133. doi: 10.3390/cancers13236133 (PMC8656724; doi:10.3390/cancers13236133)
Supplement: Supplementary file 1 [file cancers-13-06133-s001.zip › cancers-1471030-supplementary.pdf]

cancers

MDPI

Table S1. Patient characteristics of included and excluded patients (*N*=8964).

**Characteristics**	**Included patients** **N (%)**	**Excluded patients** [a] **N (%)**	***P*-value** [b]
Total number of patients	7077	1887	
Age, years			
Median age (5-95% percentiles)	70 (52-84)	73 (54-87)	<0.001
Sex			
Female	3425 (48)	919 (49)	0.814
Male	3652 (52)	968 (51)	
Histology			
Small cell lung cancer	1099 (16)	212 (11)	
Adenocarcinoma	3197 (45)	798 (42)	
Squamous cell	1424 (20)	440 (23)	<0.001
Other	1067 (15)	351 (19)	
NSCLC, otherwise unspecified	290 (4)	86 (5)	
Stage			
I	1118 (16)	586 (31)	
II	540 (8)	124 (7)	
III	1459 (21)	269 (14)	<0.001
IV	3419 (48)	733 (39)	
Unknown	541 (8)	175 (9)	
Performance status, ECOG			
0	2309 (33)	475 (25)	
1	2287 (32)	478 (25)	
2	908 (13)	271 (15)	<0.001
3 + 4	852 (12)	470 (25)	
Unknown	721 (10)	193 (10)	

NSCLC, non-small cell lung cancer; ECOG, Eastern Cooperative Oncology Group.[a]: Lung cancer patients excluded due to missing serum albumin and/or serum alkaline phosphatase measurement. [b]: P-values are calculated by the chi-square test or the nonparametric equality of medians test.
